# Radiographic evaluation of orthodontic treatment by means of four different cephalometric superimposition methods

**DOI:** 10.1590/2176-9451.20.3.029-036.oar

**Published:** 2015

**Authors:** Marcos Augusto Lenza, Adilson Alves de Carvalho, Eduardo Beaton Lenza, Mauricio Guilherme Lenza, Hianne Miranda de Torres, João Batista de Souza

**Affiliations:** 1 Full professor, Universidade Federal de Goiás (UFG), School of Dentistry, Goiânia, Goiás, Brazil; 2 Professor, Faculdades Unidas do Norte de Minas (FUNORTE)/Instituto Lenza de Pós-Graduação, Postgraduate program in Orthodontics, Goiânia, Goiás, Brazil; 3 PhD resident in Clinical Dentistry, Universidade Federal de Goiás (UFG), School of Dentistry, Goiânia, Goiás, Brazil; 4 Associate professor, Universidade Federal de Goiás (UFG), School of Dentistry, Goiânia, Goiás, Brazil

**Keywords:** Orthodontics, Growth, Radiology

## Abstract

**INTRODUCTION:**

Despite discussion on the merit of various cephalometric superimposition methods, there remains a need to assess which one can be used in daily practice with reasonably accuracy and less working time.

**OBJECTIVE:**

The aim of this study was to investigate four methods of cephalometric superimposition by means of assessing the longitudinal changes in craniofacial morphology caused by growth and response of adolescents with Class I malocclusion to orthodontic treatment involving first premolar extraction.

**METHODS:**

Pretreatment (T_1_) and post-treatment (T_2_) standardized lateral cephalometric radiographs of 31 adolescents (20 females and 11 males), with Angle Class I malocclusion and indication of premolar extraction, participated in this study. Radiographs were digitized, traced and had structures identified by means of a cephalometric software. Four superimposition methods were used: Björk structural method, Steiner/Tweed SN line, Ricketts N-Ba line at N-point and Ricketts N-Ba line at CC-point. Positional changes were quantified by horizontal and vertical linear changes in the following cephalometric landmarks: anterior/posterior nasal spine (ANS and PNS), gnathion (Gn), Gonion (Go), Pogonion (Pog), A-point and B-point. Differences between T_1_ and T_2_ in horizontal and vertical positional changes for all superimposition methods were assessed by one-way analysis of variance (ANOVA) and Bonferroni correction (p < 0.05).

**RESULTS:**

There were no statistically significant differences among the cephalometric superimposition methods or when patients' sex was considered.

**CONCLUSION:**

Björk structural method, Steiner/Tweed SN line, Ricketts N-Ba line at N-point and Ricketts N-Ba line at CC-point methods were reliable and presented similar precision when the overall facial changes due to active growth and/or orthodontic treatment were examined.

## Abstract (pt)

**INTRODUÇÃO:**

apesar dos debates sobre os méritos dos vários métodos de sobreposição cefalométrica, ainda há necessidade de se avaliar quais deles poderiam ser utilizados na prática diária, com razoável precisão e menor tempo de trabalho.

**OBJETIVO:**

o objetivo desta pesquisa foi investigar quatro métodos de sobreposição cefalométrica, avaliando as mudanças longitudinais na morfologia craniofacial causadas pelo crescimento e resposta ao tratamento, em adolescentes com má oclusão de Classe I tratados ortodonticamente com extração de primeiros pré-molares.

**MÉTODOS:**

foram usadas telerradiografias laterais pré-tratamento (T_1_) e pós-tratamento (T_2_) de 31 adolescentes (20 do sexo feminino e 11 do sexo masculino), com Classe I de Angle e indicação de extração de pré-molares. As radiografias foram digitalizadas, traçadas e as estruturas, identificadas com um *software* cefalométrico (Radiocef, Radio Memory, Belo Horizonte, Brasil). Quatro métodos de sobreposição foram utilizados para avaliar mudanças de posição devidas ao crescimento e/ou tratamento: método Estrutural de Björk; método Steiner/Tweed linha SN; Ricketts linha N-Ba no ponto N; e Ricketts linha N-Ba no ponto CC. As mudanças posicionais avaliadas pelos métodos de sobreposição foram quantificadas por mudanças lineares horizontais e verticais nos seguintes pontos cefalométricos: espinha nasal anterior (ENA), espinha nasal posterior (ENP), gnátio (Gn), Gônio (Go), Pogônio (Pog), Ponto A e Ponto B. Mudanças de posição horizontal e vertical entre T_1_ e T_2_ para todos os métodos de sobreposição foram avaliadas por análise de variância (ANOVA) e teste de Bonferroni (p < 0,05).

**RESULTADOS:**

não houve diferença estatística significativa entre os métodos de sobreposição cefalométrica em toda a amostra ou quando o sexo dos pacientes foi considerado.

**CONCLUSÃO:**

de acordo com a metodologia aplicada nesse estudo, os métodos de sobreposição cefalométrica Estrutural de Björk, de Steiner/Tweed linha SN, Ricketts linha N-Ba no ponto N e de Ricketts linha N-Ba no ponto CC foram confiáveis e com precisão semelhante para examinar as alterações faciais globais devidas ao crescimento ativo e/ou tratamento ortodôntico.

## INTRODUCTION

The need to visualize and understand the behavior of craniofacial structures in response to orthodontic treatment and continuous growth and development has motivated the emergence of several cephalometric superimposition techniques.^1^^-^ 6 Different anatomical structures, cephalometric landmarks, lines and planes of reference have been used for this purpose, allowing quantitative analysis of growth and treatment based on changes of the facial skeleton of a particular individual over a period of time.^7^^-^ 11

Cephalometric superimposition is of great importance when assessing orthodontic-orthopedic treatment response and orthognathic surgery outcomes.^11^^-^ 14 Pretreatment and post-treatment cephalometric tracings should be carefully superimposed in order to provide reliable assessment of orthodontic/growth structural changes.^13^Longitudinal changes in craniofacial morphology caused by growth and treatment response can be measured by superimposing a series of lateral cephalograms, using relatively stable landmarks, such as cranial base, cranial points, lines or regional contours, as reference.^15^

Several superimposition methods have been described in the literature. Björk and Skieller^4^^,^ 5 state-of-the-art structural superimposition method based on Björk implant studies on craniofacial growth has been used widely. Superimposition is made on specific anatomical bone structures. This method, however, relies on the quality of the radiograph, particularly with regard to optimal contrast and density. Steiner/Tweed SN line method,^2^Ricketts^6^ N-Ba line at N-point and Ricketts N-Ba line at CC-point methods have also been described in the literature. Superimposition on the cranial base provides an overall assessment of growth and treatment changes of facial structures. It will not identify specific sites of growth, but will aid assessment of the amount and direction of maxillary and mandibular growth, as well as the overall displacement of teeth and associated soft tissue changes.

Superimposition methods have revealed deficiencies or difficulties in comparison to others.^16^^,^ 17 Some studies^9^^,^ 18 demonstrate inaccuracy of cephalometric superimposition methods; while others^13^^,^ 19 ^,^ 20 suggest the use of more than one method in order to increase the procedure reliability, provide additional information and make it possible to assess sagittal skeletal and dental changes more accurately. Although some practitioners may opt to do so, it is time consuming and may not be ideal in private settings. Nevertheless, this is not to suggest that cephalometric superimposition is not a useful measurement tool used to assess the extent of dentofacial changes. Rather, studies^13^^,^ 16 ^,^ 17 indicate that it may be used with sufficient degree of accuracy for clinical diagnosis and treatment.

The most significant limitation of cephalometric superimposition is that three-dimensional changes are measured in two dimensions. The advent of cone-beam computed tomography has provided new insights of three-dimensional changes induced by normal growth and orthodontic treatment. Will it be the substitute for the traditional superimposition methods used today? Nevertheless, the present emphasis on minimizing radiation exposure prevents the use of such diagnosis resource on routine conventional orthodontic practice.^21^^,^ 22

Which superimposition method is best suited to assess changes caused by growth and/or orthodontic treatment response? Are superimposition methods equally accurate and reliable? The aim of this study was to assess four different methods of cephalometric superimposition by means of examining the results of Angle Class I treatment of growing individuals treated with upper and lower premolar extractions.

## MATERIAL AND METHODS

The present study was approved by Universidade Federal de Goiás Institutional Review Board under protocol number 055/2007.

## Sample selection

This retrospective observational study was conducted on pre (T_1_) and post (T_2_) treatment standardized lateral cephalometric radiographs of 31 adolescents (20 females and 11 males) with mean age of 13 years and 4 months at T_1_ and 17 years and 6 months at T_2_. Radiographs were obtained from the archives of Universidade Federal de Goiás, School of Dentistry, postgraduate program in Orthodontics.

Patients had been referred to orthodontic treatment due to Angle Class I malocclusion, and indication of upper and lower premolar extraction due to severe crowding or dental protrusion in permanent dentition. Only individuals presenting high-quality lateral cephalometric radiographs at the beginning and end of treatment, which allowed clear visualization of dentoskeletal structures, soft tissue and facial contour, were included. These individuals did not present any systemic conditions that could hinder the results of the study.

## Cephalometric superimposition analysis

**Figure 1.**
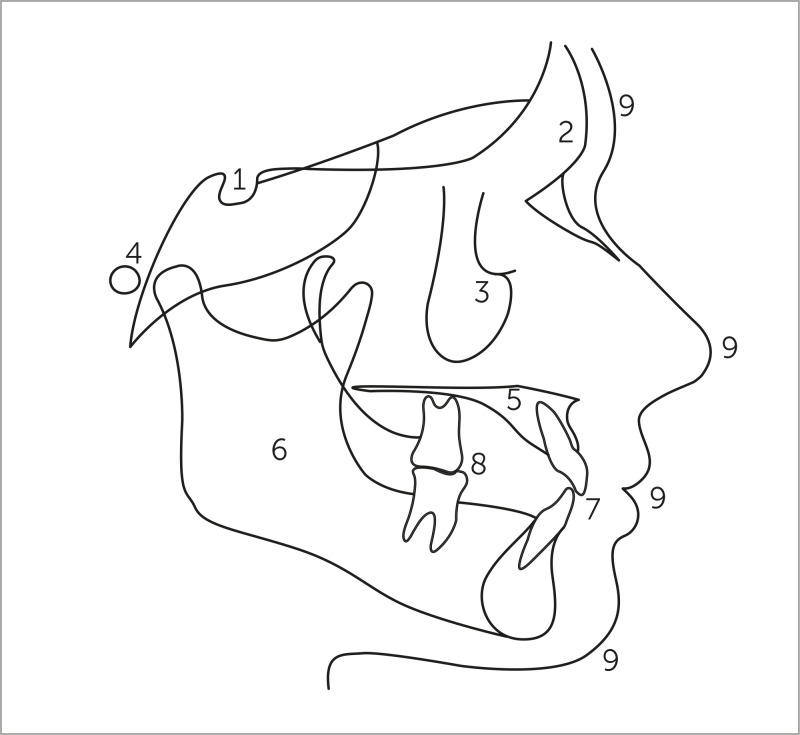
Anatomical structures and reference cephalometric landmarks used in the study; 1. sella turcica, 2. nasal and frontal bones, 3. orbit, 4. external acoustic meatus, 5. maxilla, 6. mandible, 7. upper and lower central incisors, 8. upper and lower first molars, 9. soft tissue profile.

All pre and post-treatment lateral cephalometric radiographs were digitized with a resolution of 150 dpi by means of a flatbed scanner (Hewlett-Packard Company, Palo Alto, Ca, USA) attached to a transparency reader. Images were saved in TIFF format. Each cephalogram was traced and all anatomical structures necessary for the superimposition methods were identified (Fig 1). Both tracing and superimposition procedures were performed by the same operator. Data were collected by means of Radiocef Studio 2.0 cephalometric software (Radiomemory, Belo Horizonte, Brazil).

Four superimposition methods were used to assess the positional changes caused by orthodontic treatment and associated growth, taking into account the stability of reference points, as well as their precision and visualization, and ease of the method:

- Björk structural method (M1): radiographs were superimposed on the reference bone structures in the anterior cranial base, as described by Björk and Skieller.^4^^,^ 5 Anterior contour of sella turcica wall, anterior contour of the median cranial fossa, the mean intersection point of the lower contours of the anterior clinoid processes, the inner surface of the frontal bone, contour of the cribriform plate, contours of the bilateral frontoethmoidal crests and contour of the median border of cerebral surfaces of the orbital roofs.
- Steiner/Tweed SN line method^2^^,^ 3 (M2): radiographs were superimposed on the SN line with registration at the S-point.
- Ricketts N-Ba line at N-point method^6^(M3): radiographs were superimposed on the N-Ba line with registration at the N-point.
- Ricketts N-Ba line at CC-point method^6^(M4): radiographs were superimposed on the N-Ba line with registration at the CC-point (center of the cranium) where the Ba-N plane intersects the Ptm-gnathion line.

The positional changes assessed by the superimposition methods were quantified on the basis of horizontal and vertical linear changes in the following cephalometric landmarks: anterior nasal spine (ANS), posterior nasal spine (PNS), gnathion (Gn), gonion (Go), pogonion (Pog), A-point and B-point; following the criteria described by Baumrind and Frantz.^7^^,^ 8 ^,^ 9

**Figure 2.**
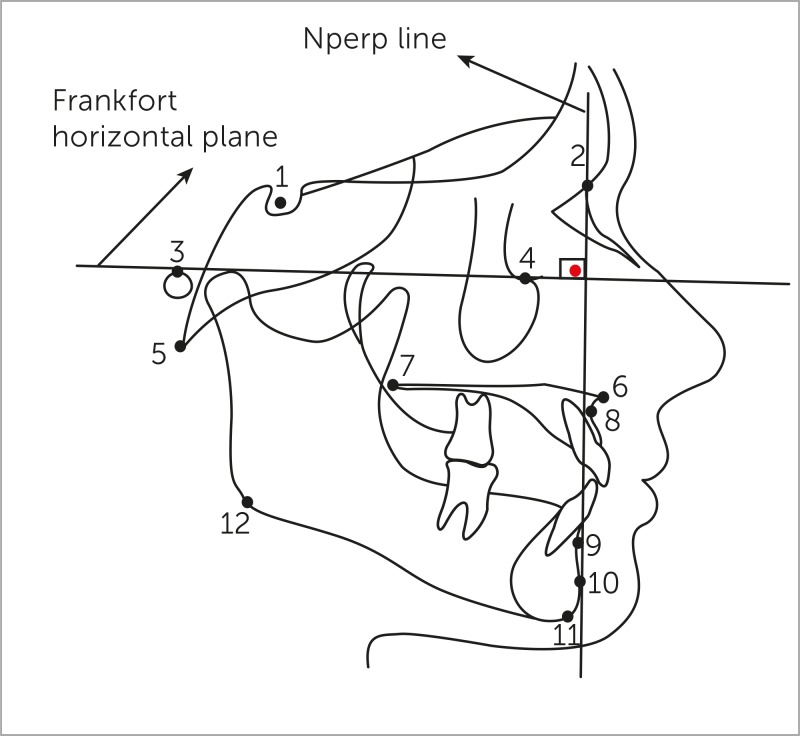
Cephalometric landmarks: 1. S (sella), 2. N (nasion), 3. Po (porion), 4. Or (orbitale), 5. Ba (basion), 6. ANS (anterior nasal spine), 7. PNS (posterior nasal spine), 8. A-point, 9. B-point, 10. Pog (pogonion), 11. Gn (gnation), 12. Go (gonion). Cephalometric planes: NPerp (nasion-perpendicular) and FHP (Frankfort horizontal plane).

Post-treatment tracings were then superimposed on pre-treatment ones so as to quantify the horizontal and vertical positional changes according to each superimposition method. Vertical alterations were measured in millimeters with a line perpendicular to Frankfort horizontal plane (FHP), whereas horizontal alterations were also measured in millimeters with a horizontal line perpendicular to Nasion (Nperp) (Fig 2). Seven horizontal measurements (ANS-Nperp, A-Nperp, PNS-Nperp, B-Nperp, Pog-Nperp, Gn-Nperp, Go-Nperp) were calculated to compare the displacement (expressed in millimeters) of the cephalometric landmarks assessed by Björk structural, Steiner/Tweed SN line, Ricketts N-Ba line at N-point and Ricketts N-Ba line at CC-point superimposition methods. Seven vertical measurements (ANS-FHP, A-FHP, PNS-FHP, B-FHP, Pog-FHP, Gn-FHP, Go-FHP) were also calculated.

A reference coordinate system was established for pre and post-treatment cephalometric radiographs. For the vertical measurements obtained by a line perpendicular to the Frankfort horizontal plane, the X-axis was used; all measurements below this axis were negative while those above it were positive. For the horizontal measurements obtained by a line perpendicular to the nasion-perpendicular (Nperp) line, the Y-axis was used; all measurements to the left of this axis were negative while those to the right were positive.

Raw numbers were registered in an Excel spreadsheet for later statistical calculations. The positional change of each landmark was then compared within this coordinate system for each superimposition method.^9^ Pre and post-treatment differences determined the horizontal and vertical linear changes of the cephalometric landmarks. 

## Error of the method

Intraexaminer reliability was determined by reassessing ten randomly selected cephalometric radiographs (five pre-treatment and five post-treatment) which were digitized, traced and measured by the same examiner twenty-one days after the first measurement. The difference between first and second cephalometric measurements was determined for each radiograph, and casual error calculated by Dahlberg^23 ^formula: E^2^ = Σ d^2^/2n, in which "d" represents the difference between the values obtained in the first and second measurements and "n" represents the number of cases in which measurements were repeated. Systematic error was calculated by paired t-test, according to Houston.^24^ Significance level was set at P **< **0.05.

## Statistical analysis

Descriptive statistics (mean and standard deviation) were calculated for all measurements obtained by the superimposition methods in both observational periods. Paired t-test was used to assess the amount of displacement according to each superimposition method on pre and post-treatment cephalometric radiographs. Differences in the amount of horizontal and vertical positional changes between T_1_ and T_2_ were assessed by one-way analysis of variance (ANOVA) and Bonferroni test for all superimposition methods. Data were analyzed by SPSS for Windows (SPSS 17.0, SPSS Inc, Chicago, III). Differences were considered statistically significant at P < 0.05.

## RESULTS

Changes caused by growth and/or orthodontic treatment, assessed by four cephalometric superimposition methods, were quantified by horizontal and vertical linear alterations in the following cephalometric landmarks: anterior nasal spine (ANS), posterior nasal spine (PNS), gnathion (Gn), gonion (Go), pogonion (Pog), A-point (subspinale) e B-point (supramentale). A total of seven horizontal and seven vertical measurements were carried out. The amount of positional changes was calculated by the difference between pre and post-treatment cephalometric measurements for each superimposition method.

The calculated reliability coefficient for examiner reliability was 0.97 for all measurements, which yields sufficient reliability. Tables 1, 2 and 3 present mean and standard deviation values (expressed in millimeters) of horizontal and vertical differences between the cephalometric measurements (difference = final value (T_2_) - initial value (T_1_)) of Björk structural (M1), Steiner/Tweed SN line (M2), Ricketts N-Ba line at N-point (M3) and Ricketts N-Ba line at CC-point (M4) superimposition methods for the whole sample, and compared according to individuals' sex. Positive values mean that the cephalometric measurement at T_2_ was greater than T_1_ - that is, the cephalometric landmark was further from the Nperp line (horizontal) or from Frankfort horizontal plane (vertical) at T_2_ in comparison to T_1_. Negative values mean that the cephalometric measurement at T_2_ was lower than T_1_ - that is, the cephalometric landmark is closer to the Nperp line (horizontal) or to Frankfort horizontal plane (vertical) at T_2_ in comparison to T_1_.

**Table 1.** Mean and standard deviation (in millimeters) of differences in horizontal (NPerp) and vertical (FHP) cephalometric measurements among Björk structural (M1), Steiner SN line (M2), Ricketts N-Ba line at N-point (M3) and Ricketts N-Ba at CC-point (M4) superimposition methods for the whole sample (n = 31).

Measure	M1	M2	M3	M4	ANOVA	M1-M2	M1-M3	M1-M4	M2-M3	M2-M4	M3-M4
ANS-Nperp	0.14 ± 3.32	0.24 ± 3.45	0.24 ± 3.40	0.21 ± 3.20	NS	-0.09 ± 0.85	-0.07 ± 0.83	-0.03 ± 0.84	0.02 ± 0.85	0.06 ± 0.86	0.04 ± 0.83
ANS-FHP	2.20 ± 4.60	2.00 ± 4.30	2.30 ± 4.50	2.40 ± 4.50	NS	0.19 ± 1.13	-0.10 ± 1.20	-0.17 ± 1.16	-0.29 ± 1.12	-0.37 ± 1.12	-0.07 ± 1.15
A-Nperp	-0.71 ± 3.60	-0.79 ± 3.50	-0.80 ± 3.50	-0.82 ± 3.50	NS	0.07 ± 0.89	0.09 ± 0.91	0.11 ± 0.90	0.01 ± 0.89	0.03 ± 0.88	0.01 ± 0.89
A-FHP	1.80 ± 2.90	1.60 ± 2.80	1.70 ± 3.00	1.70 ± 3.00	NS	0.15 ± 0.73	0.06 ± 0.75	0.08 ± 0.75	-0.08 ± 0.74	-0.14 ± 0.74	-0.05 ± 0.76
PNS-Nperp	2.90 ± 4.60	2.80 ± 4.40	3.10 ± 4.40	3.00 ± 4.40	NS	0.17 ± 0.10	-0.14 ± 1.10	-0.08 ± 1.10	-0.32 ± 1.10	-0.26 ± 1.10	0.05 ± 1.10
PNS-FHP	0.99 ± 3.50	0.62 ± 3.41	0.72 ± 3.59	0.64 ± 3.55	NS	0.36 ± 0.87	0.27 ± 0.90	0.35 ± 0.89	-0.09 ± 0.88	-0.01 ± 0.88	0.08 ± 0.90
B-Nperp	2.50 ± 3.92	2.51± 3.70	2.62 ± 3.85	2.58 ± 3.76	NS	-0.01 ± 0.96	-0.12 ± 0.98	-0.08 ± 0.97	-0.11 ± 0.96	-0.07 ± 0.94	0.04 ± 0.96
B-FHP	3.53 ± 3.58	3.52 ±3.67	3.45 ± 3.76	3.49 ± 3.66	NS	0.004 ± 0.92	0.07 ± 0.93	0.03 ± 0.92	0.07 ± 0.94	0.03 ± 0.93	-0.04 ± 0.94
Pog-Nperp	3.91 ± 3.67	3.89 ± 3.62	3.90 ± 3.62	3.81 ± 3.57	NS	0.01 ± 1.15	0.01 ± 1.15	0.09 ± 1.15	-0.05 ± 1.15	0.07 ± 1.14	0.08 ± 1.14
Pog-FHP	3.85 ± 4.00	3.66 ± 4.00	3.55 ±4.10	3.70 ± 4.06	NS	0.18 ± 1.02	0.29 ± 1.03	0.15 ± 1.02	0.11 ± 1.03	-0.03 ± 1.02	-0.14 ± 1.04
Gn-Nperp	1.07 ± 5.23	0.80 ± 5.19	0.84 ± 5.28	0.92 ± 4.97	NS	0.26 ± 1.32	0.23 ± 1.33	0.14 ± 1.30	-0.03 ± 1.33	-1.12 ± 1.29	-0.08 ± 1.30
Gn-FHP	4.78 ± 6.34	4.66 ± 6.40	4.51 ± 6.46	4.62 ± 6.38	NS	0.12 ± 1.62	0.27 ± 1.63	0.16 ± 1.62	0.15 ± 1.63	0.04 ± 1.62	-0.11 ± 1.63
Go-Nperp	1.55 ± 4.55	1.36 ± 4.48	1.47 ±4.60	1.60 ± 4.54	NS	0.18 ± 1.15	0.07 ± 1.16	-0.05 ± 1.15	-0.10 ± 1.15	-0.23 ± 2.06	-0.30 ± 1.16
Go-FHP	3.02 ± 5.93	3.03 ± 5.96	3.03 ± 5.98	2.99 ± 6.03	NS	-0.01 ± 1.51	-0.01 ± 1.51	0.02 ± 1.52	-0.02 ± 1.52	0.04 ± 1.52	0.04 ± 1.53

Values = mean ± standard deviation. Student's t-test. p < 0.05 ANOVA (one-way). Bonferroni. NS = nonsignificant.

**Table 2.** Mean and standard deviation (in millimeters) of differences in horizontal (NPerp) and vertical (FHP) cephalometric measurements among Björk structural (M1), Steiner SN line (M2), Ricketts N-Ba line at N-point (M3) and Ricketts N-Ba at CC-point (M4) superimposition methods for females (n = 20).

Measure	M1	M2	M3	M4	ANOVA	M1-M2	M1-M3	M1-M4	M2-M3	M2-M4	M3-M4
ANS-Nperp	-0.58 ± 3.28	-0.53 ± 3.29	-0.53 ± 3.30	-0.45 ± 3.20	NS	-0.05 ± 1.04	-0.13 ± 1.02	-0.03 ± 1.03	-0.08 ± 1.00	0.01 ± 1.04	0.11 ± 1.00
ANS-FHP	1.20 ± 4.22	1.14 ± 4.18	1.08 ± 4.19	1.16 ± 4.24	NS	0.05 ± 1.33	0.11 ± 1.30	0.03 ± 1.34	0.05 ± 1.32	-0.02 ± 1.33	-0.08 ± 1.33
A-Nperp	-1.20 ± 3.70	-1.20 ± 3.60	-1.10 ± 3.60	-1.10 ± 3.60	NS	0.03 ± 1.10	-0.04 ± 1.10	-0.03 ± 1.10	-0.07 ± 1.10	-0.06 ± 1.10	0.01 ± 1.10
A-FHP	1.50 ±2.90	1.60 ± 2.80	1.50 ± 2.90	1.50 ± 2.90	NS	-0.07 ± 0.90	0.07 ± 0.90	-0.02 ± 0.91	0.14 ± 0.90	0.06 ± 0.91	-0.07 ± 0.91
PNS-Nperp	2.30 ± 4.40	2.30 ± 4.40	2.30 ± 4.30	2.10 ± 4.30	NS	0.02 ± 1.40	0.05 ± 1.40	0.17 ± 1.34	0.03 ± 1.40	0.14 ± 1.40	0.11 ± 1.40
PNS-FHP	0.51± 2.87	0.43 ± 2.75	0.44 ± 3.01	0.30 ± 2.92	NS	0.06 ± 0.90	0.04 ± 0.84	0.05 ± 0.89	-0.01 ± 0.89	-0.05 ± 0.89	0.01 ± 0.89
B-Nperp	3.28 ± 2.88	3.22 ± 2.79	3.24± 2.84	3.23 ± 2.84	NS	0.06 ± 0.89	0.04 ± 0.90	0.05 ± 0.92	-0.01 ± 0.88	-0.05 ± 0.89	0.01 ± 0.85
B-FHP	3.71±3.11	3.73 ± 3.08	3.68 ± 3.20	3.73 ± 3.08	NS	-0.02 ± 0.97	0.03 ± 0.99	-0.01 ± 0.97	0.05 ± 0.99	0.05 ± 0.97	-0.04 ± 0.99
Pog-Nperp	3.91± 3.67	3.89 ± 3.62	3.90 ± 3.62	3.81 ±3.57	NS	0.01 ± 1.15	0.01 ± 1.12	-0.02 ± 1.10	0.07 ± 1.14	0.26 ± 1.29	0.08 ± 1.14
Pog-FHP	3.17 ± 4.23	3.16 ± 4.19	3.08 ± 4.26	3.16 ± 4.22	NS	0.006 ± 1.33	0.08 ± 1.34	0.004 ± 1.33	0.07 ± 1.34	-0.02 ± 1.33	-0.08 ± 1.34
Gn-Nperp	1.97 ±5.27	1.68 ± 5.23	1.64 ± 5.37	1.61 ± 4.95	NS	0.28 ± 1.66	0.32 ± 1.68	0.36 ± 1.62	0.03 ± 1.68	0.07 ± 1.61	0.03 ± 1.63
Gn-FHP	2.99 ± 4.79	3.05 ± 4.86	2.83 ± 4.87	3.00 ± 4.78	NS	-0.06 ± 1.53	-0.16 ± 1.50	-0.01 ± 1.51	0.22 ± 3.34	0.05 ± 1.53	-0.17 ± 1.53
Go-Nperp	2.25 ± 4.43	2.21 ± 4.36	2.18 ± 4.46	2.38 ± 4.27	NS	0.04 ± 1.38	0.06 ± 1.41	-0.13 ± 1.38	0.02 ± 1.40	-0.17 ± 1.36	-0.02 ± 1.38
Go-FHP	1.48 ± 4.97	1.51 ± 5.09	1.48 ± 5.09	1.42 ± 5.16	NS	-0.02 ± 1.59	-0.07 ± 1.59	0.05 ± 1.60	0.02 ± 1.61	0.08 ± 1.62	0.06 ± 1.62

Values = mean ± standard deviation. Student's t-test. p < 0.05 ANOVA (one-way). Bonferroni. NS = nonsignificant.

**Table 3.** Mean and standard deviation (in millimeters) of differences in horizontal (NPerp) and vertical (FHP) cephalometric measurements among Björk structural (M1), Steiner SN line (M2), Ricketts N-Ba line at N-point (M3) and Ricketts N-Ba at CC-point (M4) superimposition methods for males (n = 11).

Measure	M1	M2	M3	M4	ANOVA	M1-M2	M1-M3	M1-M4	M2-M3	M2-M4	M3-M4
ANS-Nperp	1.70± 3.90	1.30 ± 3.80	1.40 ± 3.80	1.30 ± 3.60	NS	0.32 ± 1.64	0.26 ± 1.64	0.36 ± 1.61	-0.06 ± 1.62	0.03 ± 1.58	0.09 ± 1.59
ANS-FHP	1.46 ± 3.10	1.65 ± 3.41	1.41 ± 3.13	1.49 ± 3.18	NS	-0.18 ± 1.39	0.05 ± 1.33	-0.02 ± 1.34	0.23 ± 1.40	1.49 ± 0.95	-0.08 ± 1.35
A-Nperp	0.09 ± 3.55	-0.05 ± 3.26	-0.23 ±3.53	-0.26 ± 3.45	NS	0.15 ± 1.45	0.33 ± 1.51	0.36 ± 1.49	0.17 ± 1.45	0.20 ± 1.43	0.02 ± 1.49
A-FHP	2.12 ± 3.18	1.57 ± 2.99	2.08 ± 3.38	2.09 ± 3.20	NS	0.54 ± 1.32	0.04 ± 1.40	0.02 ± 1.36	-0.50 ± 1.36	-0.53 ± 1.38	-0.02 ± 1.40
PNS-Nperp	4.05± 4.90	3.60 ± 4.30	4.55 ± 4.31	4.60 ± 4.37	NS	0.45 ± 1.97	-0.49 ± 1.97	-0.55 ± 1.98	-0.95 ± 1.83	-1.00 ± 1.85	-0.05 ± 1.89
PNS-FHP	1.73 ± 4.26	0.90 ± 4.31	1.14 ± 4.38	1.17 ± 4.38	NS	0.82 ± 1.75	0.590 ± 1.76	0.55 ± 1.76	0.23 ± 1.77	-0.26 ± 1.77	-0.03 ± 1.79
B-Nperp	1.07 ± 5.22	1.22 ± 4.84	1.51 ± 5.21	1.41 ± 4.97	NS	-0.14 ± 2.15	-0.43 ± 2.22	-0.34 ± 2.17	-0.28 ± 2.14	-0.19 ± 2.09	0.09 ± 2.17
B-FHP	3.19 ± 4.45	3.14 ±4.70	3.02 ± 4.75	3.06 ± 4.68	NS	0.04 ± 1.95	0.16 ± 1.96	0.13 ± 1.95	0.11 ± 2.01	0.08 ± 2.00	-0.03 ± 2.05
Pog-Nperp	-0.56± 4.96	-0.79 ± 4.94	-0.61 ± 5.01	-0.30 ± 5.01	NS	-0.82 ± 2.38	-1.00 ± 2.40	-1.03 ± 2.34	-0.21 ± 2.25	-0.22 ± 2.22	-0.02 ± 2.24
Pog-FHP	8.04 ± 7.66	7.58 ± 7.96	7.56 ± 8.01	7.57 ± 7.99	NS	0.51 ± 1.51	0.68 ± 1.54	0.42 ± 1.52	0.17 ± 1.60	-0.09 ± 1.58	-0.27 ± 1.61
Gn-Nperp	0.26± 4.69	-1.16 ± 4.50	0.17 ± 4.75	0.17 ± 4.87	NS	0.83 ± 3.70	1.07 ± 3.48	1.02 ± 3.51	0.24 ± 0.93	0.18 ± 0.94	-0.05 ± 0.34
Gn-FHP	8.04 ± 7.66	7.58 ± 9.96	7.56 ± 8.01	7.57 ± 7.99	NS	0.23 ± 2.11	0.23 ± 2.13	-0.25 ± 2.13	-0.17 ± 2.12	-0.48 ± 2.10	0.05 ± 3.41
Go-Nperp	0.26 ± 4.69	-0.16 ± 4.50	0.17 ± 4.75	0.17 ± 4.87	NS	-0.41 ± 1.38	0.02 ± 0.31	-0.05 ± 0.45	0.43 ± 1.34	0.35 ± 1.49	-0.30 ± 2.14
Go-FHP	5.82 ± 6.71	5.81 ± 6.64	5.86 ± 6.69	5.84 ± 6.70	NS	0.01 ± 2.85	-0.03 ± 2.86	-0.02 ± 2.86	-0.05 ± 2.84	-0.03 ± 2.54	0.01 ± 2.85

Values = mean ± standard deviation. Student's t-test. p < 0.05 ANOVA (one-way). Bonferroni. NS = nonsignificant.

One-way analysis of variance (ANOVA) and Bonferroni test were used to assess the mean values of each variable and, thus, indicate the positional change of all cephalometric landmarks according to each superimposition method. Paired t-test was used to assess the amount of displacement for every two superimposition methods over pre and post-treatment differences. Significance level was set at 5%.

Comparison among Björk structural, Steiner SN line, Ricketts N-Ba line at N-point and Ricketts N-Ba line at CC-point superimposition methods, with regard to positional horizontal and vertical linear changes from the cephalometric landmarks anterior nasal spine (ANS), posterior nasal spine (PNS), gnathion (Gn), gonion (Go), pogonion (Pog), A-point, B-point to NPerp line and Frankfort horizontal plane, demonstrated no statistically significant difference (P > 0.05) among the four methods of cephalometric superimposition for any one of the measurements investigated in the whole group or when individual's sex was considered.

## DISCUSSION

Serial radiographic cephalometry has been used since it was simultaneously but independently discovered by Hofrath^25^ and Broadbent^26^ in 1931, as a means of measuring craniofacial changes caused by growth and/or treatment. However, its clinical application resulted in confusion and misunderstanding. For this reason, accuracy of cephalometric measurements has always been questioned. Validity and reliability of superimposition methods have been a source of research of different studies^9^^,^ 18 investigating the magnitude of superimposition errors and the extent of potential positional changes of facial structures.^27^

The principle behind cephalometric superimposition is to compare radiographs taken at different time intervals, most often pre and post-treatment or even longer, in which radiographs taken years post-retention are included. This comparison provides the orthodontist with a general overview of growth and/or treatment outcomes through changes of the facial skeleton by comparing linear and angular measurements on serial cephalograms of the same patient. However, lateral cephalometric radiographs taken at different time intervals and by different operators are difficult to reproduce with a satisfactory degree of accuracy.

Positional changes of the anatomical structures caused by growth and/or treatment are studied by means of cephalometric radiographs superimposed to one another on structures that are considered stable over a period of time. Broadbent^1^ was the first to publish a technique for superimposition of successive cephalometric films which demonstrated a child's facial growth. Traditionally, the method of best fit has been used, meaning that bone structures that apparently do not change over time are used. It was thought that superimposition of such structures allowed growth and/or treatment changes in other skeletal structures to be demonstrated. Anterior cranial base superimposition proposed by De Coster^28^ was based on that principle. 

Other superimposition methods have been proposed. Simpler methods based on two or three easily identified cephalometric landmarks have been used. Thus, the sella-nasion line, with sella as the registering landmark, was proposed as an ideal superimposition method by Steiner^2^ and Tweed^3^. However, this method implies that sella is stable and that an increase in the SN line would be due to positional changes of the nasion. Ricketts^6^ developed a superimposition method based on the posterior region of the cranial base and the nasion-basion line, reasoning that growth of the spheno-occipital synchondrosis would play an important role until the end of puberty. Melsen^29^ found that sella is not stable during growth due to remodeling of the fossa. In addition, the author found that nasion and basion changed considerably in position, direction and the amount of growth, which made them unreliable structures on which cephalometric superimposition could be based.

Based on their implant studies, Bjork and Skieller^4^^,^ 5 identified the location of natural reference markers on the anterior cranial base, mandible and maxilla. This method is known as the structural method. In this study, superimposition on the anterior cranial base was used.

Perhaps the most important limitation of traditional cephalometric radiographic measurement is that three-dimensional changes are measured in only two dimensions. Cone-beam computed tomography certainly offers new knowledge on the changes caused by growth and/or orthodontic treatment. However, the present emphasis on minimizing radiation exposure still prevents the use of this new diagnostic modality on a routine basis in Orthodontics.^15^^,^ 16

Cephalometric superimposition has evoked much confusion and controversy.^30^ Basically, all methods of superimposition have been used through the years whereby landmarks and lines, which serve as the registration points and guides to orient images, were traced. Most often double images of bilateral structures are not consistent in serial cephalometric radiographs due to head positioning; for this reason, anatomical or structural landmarks are not consistently identifiable. Thus, different superimposition methods have different degrees of accuracy. Each one of these methods has specific limitations associated with the error of the method.

The four methods of cephalometric superimposition assessed in this study yielded acceptable results and, when compared, did not present statistically different results. Sexual dimorphism was not present when male and female groups were compared. Thus, the clinical choice of any superimposition method will provide the orthodontist with important information on growth and/or treatment outcomes. There was a high degree of reliability and precision when the cephalometric landmarks were digitally identified by Radiocef software. Intraexaminer error values after twenty-one days were similar and with no statistically significant differences in comparison to the first cephalometric measurements.

Vasconcelos et al^31 ^compared the use of Radiocef 2.0 cephalometric software to Dentofacial Planner 7.02 cephalometric software. The authors concluded that regardless of which method was used, there was significant similarity of results. Additionally, no statistically significant differences were found when digital and manual tracings were compared, which confirms the reliability of the software used in the present study. 

Some studies demonstrate^7^^,^ 8 ^,^ 9 ^,^ 32 ^,^ 33 that it is common to find errors inherent to superimposition methods, and that the orthodontist should not base his clinical decision on one method alone to interpret the effects of growth and/or orthodontic treatment. As a result, the authors suggested the use of more than one cephalometric superimposition method in order to obtain additional clinical information.^9^^,^ 10 ^,^ 21 ^,^ 22 ^,^ 34

The source of error may affect the reliability of cephalometric superimposition. Errors in cephalometry are unavoidable. They come from various sources and arise at any stage of the cephalometric procedure.^35^ Problems may arise due to the cephalometric imaging technique itself, quality of the original radiograph, operator's care and expertise, the protocol used to record superimposition, distortions, landmark identification and tracing errors, and also due to errors associated with growth and remodeling of the anatomical structure.

Stability and precision of some anatomical sites or cephalometric landmarks are a matter of great concern because there is no such thing as stable points, lines of reference or anatomical structures which would allow accurate superimposition during growth and development.^13^^,^ 36 For Baumrind, Miller and Molthen,^9^ cephalometric superimposition methods have the common objective of providing information about positional changes caused by growth and development and/or orthodontic treatment, but may also lead to different results according to the reference area used by each method. In this study, however, no statistically difference was observed among the superimposition methods used.

The superimposition methods assessed in this study are based on the relative stability of the cranial base, but used its different structures as reference. In accordance with suggestions made by Ghafari, Engel and Laster^11^as well as Sakima, Sakima and Melsen^37^ on the use of the cranial base as reference to cephalometric superimposition, this study does not suggest any degree of superiority among Björk structural, Steiner/Tweed SN Line, Ricketts N-Ba line at N-point and Ricketts N-Ba line at CC-point superimposition methods. The four methods of cephalometric superimposition assessed in this study yielded acceptable results, leading the practitioner to select the method with which he/she is most familiar.

## CONCLUSION

The results of this study suggest that Björk structural, Steiner SN Line, Ricketts N-Ba line at N-point and Ricketts N-Ba line at CC-point superimposition methods are equally reliable and present a similar degree of accuracy to demonstrate horizontal and vertical positional changes, thereby allowing the orthodontist to perform an overall assessment of the effects occurring due to growth and/or orthodontic treatment.
